# Resistance to extended-spectrum cephalosporins in *Escherichia coli* and other *Enterobacterales* from Canadian turkeys

**DOI:** 10.1371/journal.pone.0236442

**Published:** 2020-09-14

**Authors:** Jonathan Moffat, Gabhan Chalmers, Richard Reid-Smith, Michael R. Mulvey, Agnes Agunos, Julie Calvert, Ashley Cormier, Nicole Ricker, J. Scott Weese, Patrick Boerlin

**Affiliations:** 1 Department of Pathobiology, Ontario Veterinary College, University of Guelph, Guelph, Ontario, Canada; 2 Centre for Foodborne, Environmental and Zoonotic Infectious Diseases, Public Health Agency of Canada, Guelph, Ontario, Canada; 3 National Microbiology Laboratory, Public Health Agency of Canada, Winnipeg, Manitoba, Canada; Institut National de la Recherche Agronomique, FRANCE

## Abstract

The goal of this study was to determine the frequency of resistance to extended-spectrum cephalosporins (ESCs) in *Escherichia coli* and other *Enterobacterales* from turkeys in Canada and characterize the associated resistance determinants. Pooled fecal samples were collected in 77 turkey farms across British Columbia, Québec, and Ontario. Isolates were obtained with and without selective enrichment cultures and compared to isolates from diagnostic submissions of suspected colibacillosis cases in Ontario. Isolates were identified using MALDI-TOF and susceptibility to ESCs was assessed by disk diffusion. The presence of *bla*_CMY_, *bla*_CTX-M_, *bla*_TEM,_ and *bla*_SHV_ was tested by PCR. Transformation experiments were used to characterize *bla*_CMY_ plasmids. Genome sequencing with short and long reads was performed on a representative sample of *bla*_CTX-M_-positive isolates to assess isolates relatedness and characterize *bla*_CTX-M_ plasmids. For the positive enrichment cultures (67% of total samples), 93% (587/610) were identified as *E*. *coli*, with only a few other *Enterobacterales* species identified. The frequency of ESC resistance was low in *E*. *coli* isolates from diagnostic submission (4%) and fecal samples without selective enrichment (5%). Of the ESC-resistant *Enterobacterales* isolates from selective enrichments, 71%, 18%, 14%, and 8% were positive for *bla*_CMY_, *bla*_TEM,_ *bla*_CTX-M,_ and *bla*_SHV_, respectively. IncI1 followed by IncK were the main incompatibility groups identified for *bla*_CMY_ plasmids. The *bla*_CTX-M-1_ gene was found repeatedly on IncI1 plasmids of the pMLST type 3, while *bla*_CTX-M-15_, *bla*_CTX-M-55_, and *bla*_CTX-M-65_ were associated with a variety of IncF plasmids. Clonal spread of strains carrying *bla*_CTX-M_ genes between turkey farms was observed, as well as the presence of an epidemic *bla*_CTX-M-1_ plasmid in unrelated *E*. *coli* strains. In conclusion, *Enterobacterales* resistant to ESCs were still widespread at low concentration in turkey feces two years after the cessation of ceftiofur use. Although *bla*_CMY-2_ is the main ESC resistance determinant in *E*. *coli* from Canadian turkeys, *bla*_CTX-M_ genes also occur which are often carried by multidrug resistance plasmids. Both clonal spread and horizontal gene transfer are involved in parallel in the spread of *bla*_CTX-M_ genes in *Enterobacterales* from Canadian turkeys.

## Introduction

The World Health Organization (WHO) and the World Organisation for Animal Health (OIE) define extended-spectrum cephalosporins (ESCs) as critically important antimicrobial agents [1, 2]. One member of this class, ceftiofur, was used routinely in an extra-label manner in Canada for the prevention of colibacillosis in poultry until 2014, when it was voluntarily withdrawn by the industry [3, 4]. The turkey industry has also been phasing out the preventative use of other important antimicrobials [3, 4] and the Veterinary Drugs Directorate of Health Canada has changed antimicrobial use policies in order to further reduce their use in animals and veterinary medicine [5].

The main ESC resistance mechanisms involve the production of extended spectrum β-lactamases (ESBL) and AmpC β-lactamases. Among the latter, CMY-2, a typically plasmid-mediated AmpC β-lactamase, is the dominant variant in North America. In Canada, CMY-2 was first detected in farm animals in *Salmonella enterica* in 1995 [6]. The associated *bla*_CMY-2_ gene has been found since on a variety of *S*. *enterica* and *E*. *coli* plasmids in Canadian farm animals, including plasmids of the FIB, I1, A/C, K, K/B, and B/O incompatibility groups [7–10]. Until recently, CMY-2 has been the main determinant of ESC resistance in bacteria from farm animals in Canada [11, 12] and the few ESBLs found were SHV-2 and SHV-2a [13]. Although CTX-Ms were detected in human and canine clinical isolates in Canada in the early 2000’s [14, 15], they have appeared only recently in *Enterobacterales* from farm animals in Canada [9, 12]. They have now spread among bacteria from cattle, chicken, and swine [9, 10, 11, 16, 17]. A variety of CTX-M types have been identified in *E*. *coli* from cattle and swine [12, 16] but those from chickens have all been CTX-M-1 and typically located on IncI1 plasmids [9, 10, 16].

In contrast with other food animal species and despite the economic importance of turkey production and potential role in the epidemiology of ESC resistance, little research has been done to identify the genetic determinants of ESC resistance in bacteria from turkeys. Therefore, this study aimed to fill this gap and investigated ESC resistance in fecal *Enterobacterales* and specifically in *E*. *coli* and *S*. *enterica* from diagnostic submissions from Canadian turkey. The diversity of ESC resistance plasmids was further assessed for a subset of isolates by a variety of methods, including transformation, replicon typing, and whole genome sequencing.

## Materials and methods

### Fecal sample and bacterial isolate collection

Three sets of isolates were used for this study. The first one including *E*. *coli* and other *Enterobacterales* species was obtained from fecal samples using enrichment broth and plates containing ESCs. The second set including both *E*. *coli* and *S*. *enterica* was obtained without ESC selection from the same fecal samples. The third set included both *E*. *coli* and *S*. *enterica* from diagnostic submissions.

The pooled fecal samples used to obtain the first two sets of isolates were collected by the Canadian Integrated Program for Antimicrobial Resistance Surveillance (CIPARS) between May 2016 and June 2017 from 77 sentinel turkey farms in the Canadian provinces of Ontario, Québec, and British Columbia [18]. Four 25 g pooled samples were collected per farm, one from each quadrant of a barn. Each sample was diluted 1:10 in Buffered Peptone Water (Becton, Dickinson and Company, Franklin Lakes, NJ, USA) and frozen in aliquots at -70°C after mixing with equal amounts of Brucella Broth (Becton, Dickinson and Company) containing 15% glycerol until further use. Cultures with selective media were performed to obtain the first set of isolates from these frozen samples as described previously [16]. Briefly, the equivalent of 90 mg of feces were diluted in 18 mL of Enterobacteriaceae enrichment broth (Becton, Dickinson and Company) containing 2 mg/L of cefotaxime and incubated overnight at 37°C with shaking. Ten μL of these cultures were streaked on MacConkey agar plates (Becton, Dickinson and Company) supplemented with 1 mg/L ceftriaxone and incubated overnight at 37°C. Three isolates were systematically selected per plate: any non-mucoid, lactose-fermenting colonies were selected first followed by any mucoid, lactose-fermenting colonies, if present. Non lactose-fermenting isolates, if present, were the tertiary priority for sampling other types of colonies. In parallel to these enrichment cultures, CIPARS isolated one generic *E*. *coli* and *S*. *enterica*, when present, from each of the samples mentioned above using standard protocols without antimicrobial-containing media [18]. These *E*. *coli* and *S*. *enterica* isolates were tested for susceptibility to the standard CIPARS panel of antimicrobial agents [18]. They were included in the second set of isolates for further characterization when they had reduced susceptibility to ceftriaxone (MIC > 1 μg/ml) or cefoxitin (MIC > 8 μg/ml). The third set of isolates consisted of all *E*. *coli* isolates from turkey colibacillosis cases and *Salmonella* isolates recovered from turkey diagnostic submissions (one per submission) at the Animal Health Laboratory of the University of Guelph between October 2015 and April 2017. Isolates from this third set were processed further for antimicrobial susceptibility testing and genotyping in the same way as those from the first set.

### Isolate identification and antimicrobial susceptibility testing

Isolates from selective enrichment cultures were screened using oxidase strips (Sigma-Aldrich, St. Louis, MO, USA) and catalase tests (in-house reagent). Oxidase-negative and catalase-positive isolates were identified to the species level using MALDI-TOF mass spectrometry (Bruker Daltonik GmbH, Bremen, Germany) before further characterization. Isolates were tested for susceptibility to cefoxitin, ceftazidime, cefotaxime, cefotaxime plus clavulanic acid and ertapenem, using the disk diffusion method following the Clinical Laboratory Standards Institute’s performance standards [19, 20]. Only isolates with inhibition zone diameters below the resistance breakpoint were included as resistant.

### PCR screening for ESC resistance genes

All isolates were screened for the genes *bla*_CMY_, *bla*_CTX-M_, *bla*_SHV_, and *bla*_TEM_ using single and multiplex PCR as previously described [21, 22]. Lysates were prepared using the boiling method, centrifuged, and the supernatants were used as PCR templates. Negative controls were made with each batch of lysates and run alongside test samples. Isolates known to have the genes of interest and confirmed by DNA sequencing were used as positive controls in each PCR. Sanger sequencing of PCR products at the University of Guelph Laboratory Services was used to determine the gene variants in isolates where either *bla*_SHV_ or *bla*_TEM_ was the only putative ESC resistance gene detected (see Table 1).

**Table 1:** PCR primers for detection and sequencing of AMR genes.

Gene	Primer	Sequence	Amplicon size	Reference
*bla_TEM_*	TEM-F	TTCTTGAAGACGAAAGGGC	1150	[23]
	TEM-R	ACGCTCAGTGGAACGAAAAC		
*bla_CMY_*	CMYF	GACAGCCTCTTTCTCCACA	1000	[21]
	CMYR	TGGACACGAAGGCTACGTA		
*bla_SHV_*	blaSHVextFC-OPT	GGTTATTCTTATTTGTCGCTTCTT	913	[21]
	blaSHVextRC-OPT	TACGTTACGCCACCTGGCTA		
	shvcolom-F	AGGATTGACTGCCTTTTTG	393	[21]
	shvcolom-R	ATTTGCTGATTTCGCTCG		
*bla_CTX-M_*	CTX-M-F	ATGTGCAGYACCAGTAA	512	[22]
	CTX-M-R	CCGCTGCCGGTYTTATC		

### Transformation and characterization of CMY plasmids

Formal random selection (random number generation) was used to select one *bla*_CMY_-positive isolate from an ESC selective enrichment from each of eighteen randomly selected positive farms for further characterization by Sanger sequencing of the *bla*_CMY_ gene. Transformation of the *bla*_CMY_ plasmid of these isolates was performed using a plasmid mini kit (QIAGEN, Hilden, Germany) and ElectroMAX DH10B competent cells (Invitrogen, Thermo Fisher Scientific, Carlsbad, CA) according to manufacturer’s instructions. Transformants were selected on LB agar (Becton, Dickinson and Company) supplemented with 1 mg/L of ceftriaxone. Their plasmids were prepared using the plasmid mini-kit, then run on gel electrophoresis to confirm transfer of a single plasmid. Transformants were also tested using CIPARS’s Sensititre panel for the antimicrobials amoxicillin/clavulanic acid, ampicillin, azithromycin, cefoxitin, ceftriaxone, chloramphenicol, ciprofloxacin, gentamicin, meropenem, nalidixic acid, streptomycin, sulfisoxazole, tetracycline, and trimethoprim/sulfamethoxazole [18]. Replicon types of the transferred CMY plasmids were determined by PCR as previously described [24].

### Conjugation

The transfer of *bla*_CTX-M-65_ plasmids was studied in a conjugation experiment following a previously established protocol [25] carried out on LB agar (Becton, Dickinson and Company) and in LB broth overnight at both 37°C and room temperature. The *bla*_CTX-M-65_-positive isolates, 98.1 (this study), and FC-471 [12] were used as donor strains. The nalidixic acid-resistant strain 711-nal [26] was used as a recipient. Transconjugants were selected on LB agar containing 50 mg/L nalidixic acid and 4mg/L ceftriaxone. Isolate PA3, which contains a transferable tetracycline resistance plasmid, was used as a positive control. PA3 transconjugants were selected on LB agar containing 50 mg/L nalidixic and 16 mg/L tetracycline.

### Genome sequencing and plasmid analysis

The only *bla*_CTX-M_-positive diagnostic isolate and one *bla*_CTX-M_-positive *E*. *coli* per farm in 16 farms were selected using a random number generator for further analysis using the Illumina Nextseq platform (PE150; Illumina, San Diego, CA, USA) at the National Microbiology Laboratory in Winnipeg, Manitoba. The EpiCentre MasterPure DNA Purification kit (EpiCentre, Madison, WI, USA) was used for DNA preparation following the manufacturer's instructions.

Sequence assembly was performed using the WGS Tools application for BioNumerics v7.6 (Applied Maths, Sint-Martens-Latem, Belgium) using the SPAdes *de novo* assembler, and assembly-free and assembly-based allele calling. *E*. *coli* isolates were assigned a multi-locus sequence type using the cgMLST application for BioNumerics and the BioNumerics *Escherichia coli*/*Shigella* Enterobase scheme. AMR genes were identified using the *E*. *coli* functional genotyping plugin for BioNumerics which uses ResFinder [27] from the Center for Genomic Epidemiology, Technical University of Denmark, DTU.

Long read sequencing of isolates was performed using a MinION device (Oxford Nanopore Technologies, Oxford, United Kingdom) on all *bla*_CTX-M_-positive isolates that were also sequenced using short reads. DNA purification was carried out as described above for Illumina sequencing. Sequencing libraries and barcoding preparation was done using the SQK-LSK109 and EXP-NBD104/114 ligation and native barcoding kits (Oxford Nanopore Technologies) according to the manufacturer's instructions. Two flow cells (version FLO-MIN106 R9.4) were used, and run for 48h each. Basecalling of fast5 files and demultiplexing was performed using Guppy Basecaller v3.3 (Oxford Nanopore Technologies) with barcode trimming enabled. Hybrid assembly of short and long reads was performed using Unicycler v0.4.4 [28] and visualized with Bandage v0.8.1 [29]. Assemblies were also generated with Canu v1.8 [30] and Flye v2.6 [31] and subsequently compared to unicycler assemblies. Read mapping was also performed using Geneious v9.1.7 (Biomatters, Auckland, New Zealand) to select the best assembly. If Unicycler was unsuccessful at assembling complete plasmids, Flye assemblies were polished with short reads using Racon v1.4.0 [32] and Pilon v1.23 [33]. Any *bla*_CTX-M_-bearing plasmid found in more than one isolate was aligned using the Geneious Mauve plugin v2.3.1 and annotated in BioNumerics using myRAST v36 [34].

Core genome sequence analysis was performed using the BioNumerics v7.6 (Applied Maths, Sint-Martens-Latem, Belgium) whole-genome sequencing application. Assembly-based allele calling (SPAdes algorithm) and assembly-free calling were used to determine sequence types, with the *E*. *coli*/*Shigella* Enterobase scheme. A minimum spanning tree was generated using the core genome MLST (cgMLST) data, with 1000x bootstrap resampling support. A core SNP analysis was performed for all 17 assemblies using Snippy v4.4.5 (https://github.com/tseemann/snippy), with the *E*. *coli* K-12 genome [35] used as a reference genome. The core SNP alignment output file was then used in BioNumerics to create an unrooted Neighbor Joining cluster analysis tree.

## Results

### Species identification and distribution of resistance

#### Isolates from fecal enrichment cultures

Three hundred and eight fecal samples were enriched for ESC-resistant *Enterobacterales* and one or more ESC-resistant isolate was obtained from 67% of them (82% of farms). The vast majority of isolates were *E*. *coli* in addition to a few *Klebsiella pneumoniae*, *Proteus mirabilis* and *Enterobacter cloacae*. The species distribution of positive isolates, samples, and farms is shown in Table 2. Three isolates (one *K*. *pneumoniae*, one *E*. *cloacae*, and one *P*. *mirabilis*) had intermediate resistance to ertapenem. PCRs with these three isolates for the carbapenemase genes NDM, KPC, VIM and IMP [36] were all negative.

**Table 2:** Species distribution of ESC-resistant *Enterobacterales* isolates from selective enrichment cultures.

Species	Isolates (n = 610)^a^	Samples (n = 308)^a^	Farms (n = 77)^a^
*Escherichia coli* ^b^	570 (93%)	201 (65%)	59 (77%)
*Klebsiella pneumoniae* ^b^	25 (4%)	14 (5%)	8 (10%)
*Proteus mirabilis* ^b^	8 (1%)	4 (1%)	3 (4%)
*Enterobacter cloacae* ^b^	7 (1%)	5 (2%)	2 (3%)
Total positive ^b^	610	207 (67%)	63 (82%)

^a^The numbers in parentheses represent the number of tested isolates, samples, and farms, respectively.

^b^Percentages in parentheses represent the proportion of positive isolates, samples and farms, respectively.

The distribution of ESC resistance genes among isolates, samples and farms is shown in Table 3. The *bla*_CMY_ gene was the most frequent, followed by *bla*_TEM_ and *bla*_CTX-M_, while *bla*_SHV_ was the least frequently detected. A total of 49 ESC-resistant isolates were negative for all four target genes. Of these, 27 and 22 had an AmpC or ESBL phenotype, respectively. For all isolates with an ESBL phenotype and only either *bla*_TEM_ (two *E*. *coli*) or *bla*_SHV_ (five *E*. *coli* and two *K*. *pneumoniae*), these genes were sequenced. The *bla*_TEM_ genes from *E*. *coli* were both the non-ESBL *bla*_TEM-1B_ variant. Of the five *bla*_SHV_ from *E*. *coli*, four were *bla*_SHV-2_ and one was *bla*_SHV-2a_, while both *K*. *pneumoniae* carried the ESBL gene *bla*_SHV-148_. In contrast to *E*. *coli*, *bla*_CMY_ was not commonly found in *K*. *pneumoniae* (1/25, 4%). However, *bla*_CTX-M_ was found in eight (32%) of these 25 isolates (6 samples, 4 farms). Neither *bla*_CTX-M_ nor *bla*_SHV_ was detected in either *E*. *cloacae* or *P*. *mirabilis* and all isolates of these species presented an AmpC β-lactamase phenotype.

**Table 3:** Distribution of antimicrobial resistance genes in *Enterobacterales* isolates from selective enrichment cultures.

Antimicrobial resistance genes	Bacterial species	Isolates (n = 610)^a^	Samples (n = 308)^a^	Farms (n = 77)^a^
*bla*_CMY_^b^	*E*. *coli*	426 (75%)	160 (80%)	54 (92%)
	All^c^	434 (71%)	170 (55%)	55 (71%)
*bla*_TEM_^b^	*E*. *coli*	96 (17%)	52 (26%)	24 (41%)
	All^c^	107 (18%)	58 (19%)	26 (34%)
*bla*_CTX-M_^b^	*E*. *coli*	64 (11%)	35 (17%)	15 (25%)
	All^c^	87 (14%)	42 (14%)	19 (25%)
*bla*_SHV_^b^	*E*. *coli*	24 (4%)	15 (7%)	8 (14%)
	All^c^	48 (8%)	30 (10%)	15 (19%)

^a^The numbers in parentheses represent the number of tested isolates, samples, and farms, respectively.

^b^Percentages in parentheses represent the proportion of positive isolates, samples and farms, respectively.

^c^Include *E*. *coli*, *K*. *pneumoniae*, *E*. *cloacae*, *P*. *mirabilis*.

#### CIPARS generic isolates

The *bla*_CMY_ and *bla*_SHV_ genes were detected in two and five of the ten generic *E*. *coli* isolates from the CIPARS collection resistant to ESCs, respectively. The same variant (*bla*_SHV-2a_) was identified by sequencing of PCR products in all five *bla*_SHV_-positive isolates. Neither *bla*_CTX-M_ nor *bla*_TEM_ was detected among the generic isolates from CIPARS. Only one ESC resistance gene was detected in each isolate. The three isolates with no ESC resistance gene detected had an AmpC β-lactamase phenotype. Six ESC-resistant *S*. *enterica* isolates were recovered by CIPARS from the pooled fecal samples (three serovar Indiana, two serovar Agona, and one serovar Bredeney). All were *bla*_CMY_-positive.

#### Isolates from diagnostic submissions

Six of the 160 *E*. *coli* (4%) from AHL diagnostic submissions were resistant to ESCs. Of these, five were positive for *bla*_CMY_ and one for *bla*_CTX-M_. No *bla*_SHV_ was detected among them. Only one ESC-resistant *S*. *enterica* was detected in diagnostic submissions (serovar Indiana). Similar to the CIPARS isolates, it was positive for *bla*_CMY_.

### CMY variants and CMY-plasmid characterization

Sequencing of PCR products showed that all 18 randomly selected *bla*_CMY_-positive isolates from selective enrichment cultures carried a *bla*_CMY-2_ gene. Replicon typing on the corresponding transformants showed that *bla*_CMY-2_ was located on IncI1 plasmids in 13 of them and on IncK plasmids in the remaining five. Susceptibility testing of the transformants showed that 15 of these plasmids (twelve IncI1 and three IncK) did not encode resistances other than those mediated by *bla*_CMY-2_, while one IncI1 and two IncK plasmids additionally encoded resistance to gentamicin and sulfonamides.

### Genome analysis of CTX-M-positive isolates

#### Strain diversity and CTX-M variants

One *bla*_CTX-M_-positive *E*. *coli* isolate per positive farm (three of the 19 positive farms in Table 3 had only positive *K*. *pneumoniae* and no *E*. *coli*) and the single *bla*_CTX-M_-positive isolate from diagnostic submissions were sequenced using both short and long read sequencing techniques. All sequences have been deposited in GenBank under BioProject ID PRJNA596173. The main characteristics of these 17 strains and associated CTX-M plasmids are listed in Table 4 and their similarities with the most closely related plasmids found on GenBank are listed in Table 5. The plasmids were associated with nine different STs, of which ST10 (n = 4) and ST117 (n = 3) were the most frequent. They carried four different *bla*_CTX-M_ variants. The most frequent variants were *bla*_CTX-M-1_ (n = 7) and *bla*_CTX-M-55_ (n = 6). The *bla*_CTX-M_ genes were generally plasmid-borne and were chromosomal in only two isolates (one *bla*_CTX-M-1_ and one *bla*_CTX-M-15_). Both were associated with a tryptophan synthase gene and recombinase gene, but no plasmid-related sequence was detected beyond these. No transposon or insertion sequence was detected within 100 kbp or the chromosomal *bla*_CTX-M-15_, while the chromosomal *bla*_CTX-M-1_ region was flanked by an IS6-292 on each side. The genetic environment of these two chromosomal *bla*_CTX-M_ genes was not analyzed further.

**Table 4:** Sequence type of *bla*_CTX-M_-positive *E*. *coli* isolates and characteristics of their CTX-M-plasmids.

Isolate	Sequence type	CTX-M subtype	CTX-M plasmid Inc type	CTX-M plasmid size (bp)	Other AMR genes on CTX-M plasmid	pMLST
34.1	ST10	1	N.A.	Chromosomal ^a^	N.A.	N.A.
276.2	ST117	1	IncI1	110,468 ^b^	*sul2*	3
56.2	ST117	1	IncI1	107,524 ^b^	*sul2*, *tet*(A)	3
181.1	ST117	1	IncI1	111,912 ^a^	*sul2*, *tet*(A)	3
248.3	ST115	1	IncI1	110,215 ^a^	*sul2*, *tet*(A)	3
64.1	ST3258	1	IncI1	122,123 ^a^	*aac(3)-VIa*, *aadA1*, *sul1*, *tet*(A)	Novel
81.1	ST3258	1	IncI1	122,124 ^a^	*aac(3)-VIa*, *aadA1*, *sul1*, *tet*(A)	Novel
43.3	ST4981	15	N.A.	Chromosomal ^a^	N.A.	N.A
162.2	ST206	15	IncFIB(K)	99,943 ^b^	*tet*(A), *tet*(M), *floR*, *sul2*, *dfrA*, *strA/B*, *qnrS1*, *bla*_TEM-1_	Unknown
136.2	ST58	55	IncFIA/IncFIB(AP001918)/IncFII	138,933 ^b^	*aac(3)-IId*, *qnrS1*, *tet*(A)	F31:A4:B1
268.2	ST58	55	IncFIA/IncFIB(AP001918)/IncFII	138,915 ^a^	*aac(3)-IId*, *qnrS1*, *tet*(A)	F31:A4:B1
101.3	ST10	55	IncFIB(AP001918)/IncFIC(FII)	116,752 ^b^	*aph(3')-Ia*, *aac(3)-IId*, *tet*(A) ^*c*^	F18:A-:B1
28.1	ST227	55	IncFIB(AP001918)/IncFIC(FII)	132,062 ^a^	*aph(3')-Ia*, *aadA1*, *aac(3)-IId*, *floR*, *sul3*, *tet*(A) ^*c*^	F18:A-:B1
176.1	ST10	55	IncFIB(AP001918)/IncFIC(FII)	132,058 ^a^	*aph(3')-Ia*, *aadA1*, *aac(3)-IId*, *floR*, *sul3*, *tet*(A) ^*c*^	F18:A-:B37
154AHL	ST10	55	IncFIB(AP001918)/IncFIC(FII)	132,199 ^b^	*aph(3')-Ia*, *aadA1*, *aac(3)-IId*, *floR*, *sul3*, *tet*(A) ^*c*^	F18:A-:B1
98.1	ST683	65	IncFIA(HI1)/IncR	99,596 ^a^	*aadA1*,*bla*_OXA-10_, *qnrS1*, *floR*, *cmlA*, *ARR-2*, *tet*(A), *dfrA*	F-:Anew:B-
202.1	ST683	65	IncFIA(HI1)/IncR	99,589 ^a^	*aadA1*,*bla*_OXA-10_, *qnrS1*, *floR*, *cmlA*, *ARR-2*, *tet*(A), *dfrA*	F-:Anew:B-

^a^ Assembled using Unicycler.

^b^ Assembled using Flye and polished with Pilon.

^c^ These plasmids also carried the colicin M gene *cma*, as well as the colicin B gene *cba* and both inhibitor genes *cmi* and *cbi*.

**Table 5:** List of plasmid sequences available on GenBank most similar to the *bla*_CTX-M_ plasmids of the present study.

Plasmid from this study			Most similar plasmid					
CTX-M subtype		Isolate	Plasmid / Bacterial species	Accession number^a^	Coverage	Identity	*bla*_CTX-M_ variant	Source / Country of origin
1	276.2		pCOV28A / *E*. *coli*	MG649027.1	92%	99.98%	1	Chicken environment / France
1	56.2		pTC_N40607 / *E*. *coli*	CP007651.1	97%	99.99%	1	Cattle / USA
1	181.1		pCOV11 / *E*. *coli*	MG648913.1	96%	100.00%	1	Chicken / France
1	248.3		p369 / *E*. *coli*	KT779550.1	98%	99.99%	1	Chicken / France
1	64.1		Unnamed plasmid	CP024285.1	89%	99.99%	none	Unknown / USA
1	81.1		Unnamed plasmid	CP024285.1	89%	99.99%	none	Unknown / USA
15	162.2		pPGRT46 / *E*. *coli*	KM023153.1	91%	99.95%	15	Human / Nigeria
55	136.2		pN16EC0879-1 / *E*. *coli*	CP043745.1	100%	99.93%	55	Ground turkey / USA
55	268.2		pN16EC0879-1 / *E*. *coli*	CP043745.1	100%	99.95%	55	Ground turkey / USA
55	101.3^b^		pMCR1-PA / *E*. *coli*	CP029748.1	96%	99.93%	14, 55	Unknown / USA
55	28.1^b^		pMCR1-PA / *E*. *coli*	CP029748.1	91%	99.98%	14, 55	Unknown / USA
55	176.1^b^		pMCR1-PA / *E*. *coli*	CP029748.1	92%	99.94%	14, 55	Unknown / USA
55	154AHL^b^		pMCR1-PA / *E*. *coli*	CP029748.1	92%	99.94%	14, 55	Unknown / USA
65	98.1		pCTXM-2248 / *E*. *coli*	MG836696.1	77%	99.67%	14	Unknown / China
65	202.1		pCTXM-2248 / *E*. *coli*	MG836696.1	77%	99.80%	14	Unknown / China

^a^ Accession number of the plasmid sequence on GenBank most similar to the corresponding plasmid from this study.

^b^ Contrary to the pMCR1-PA plasmid, the plasmids from this study did not carry any *mcr* gene.

**Fig 1:**
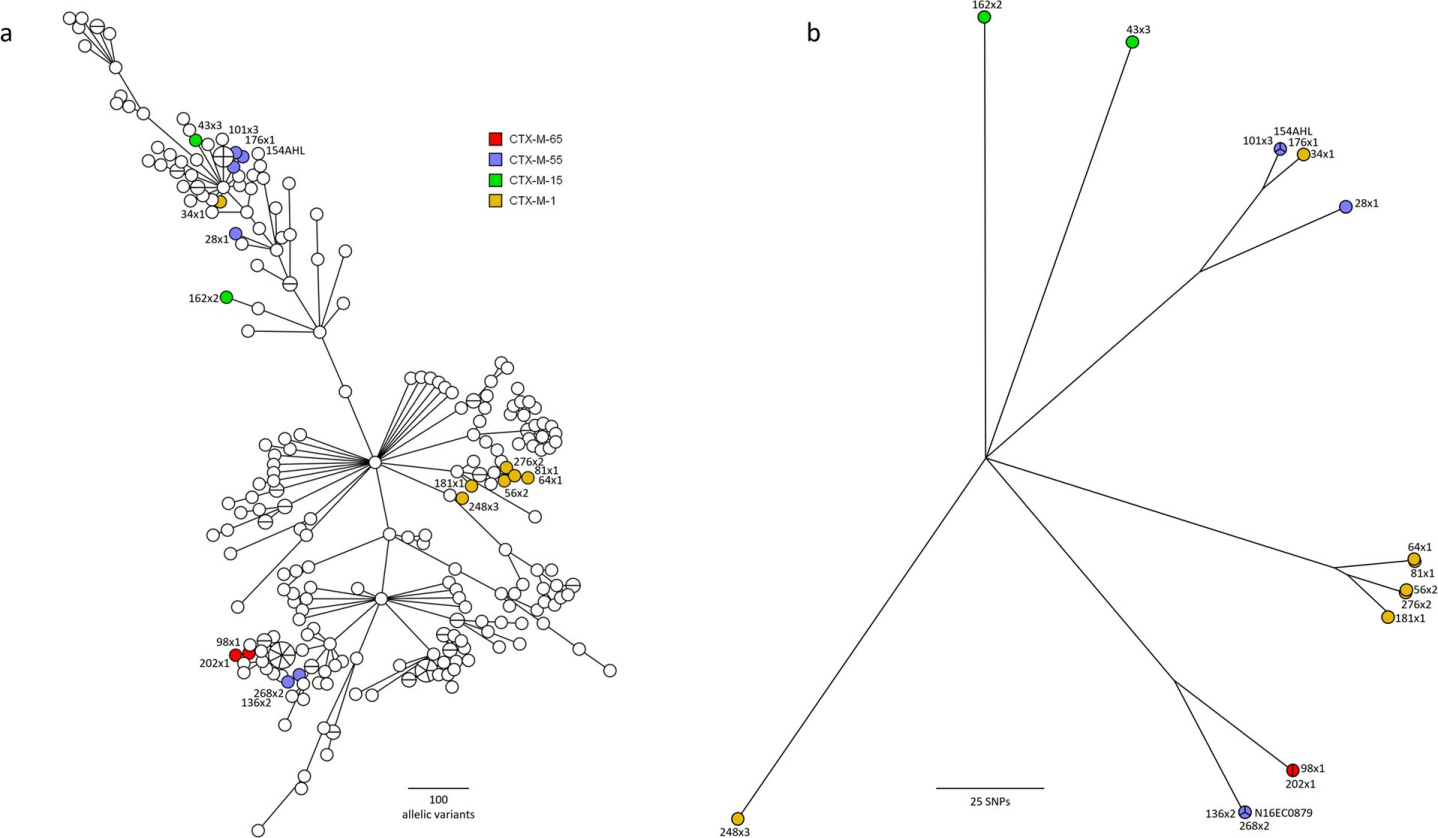
Genetic relationships between *bla*_CTX-M_-positive isolates based on core genome analysis. **a:** Minimum spanning tree based on core genome MLST analysis including 268 *bla*_CTX-M_-positive isolates from animals in Canada. **b:** Unrooted neighbor joining tree based on core SNP analysis of the 17 sequenced isolates from the present study, using *E*. *coli* K-12 as a reference genome. Isolates from the present study are labelled with colours, according to CTX-M subtypes. Isolates are identified by the same code as in Table 4 and Chr indicates chromosomal location of the *bla*_CTX-M_ genes. N16EC0879 is from an isolate obtained from ground turkey in the USA.

The *bla*_CTX-M-1_ and *bla*_CTX-M-55_ genes were found in four and three STs, respectively, and both genes were found among ST10 isolates. The putative genetic relationships between isolates derived from core genome MLST (2,513 loci) including 268 CTX-M-positive *E*. *coli* isolates from different animal sources in Canada [16] are depicted in Fig 1A. The core SNP (139,135 bp) analysis showed pair-wise differences ranging from 23 bp to 78,109 bp (median 65,620 bp). The putative relationships of the isolates from this study derived from core SNPs are presented in Fig 1B.

The cgMLST minimum spanning tree and the neighbor joining tree based on SNP analysis both show some loose clustering of isolates with similar *bla*_CTX-M_ variants (Fig 1). However, this is not a consistent trend. Two main clusters can be seen for the isolates carrying *bla*_CTX-M-55_ and one of the *bla*_CTX-M-1_ isolates (248.3) is clearly distinct from the others. The two isolates with chromosomal *bla*_CTX-M_ genes are clearly unrelated to those with the same plasmid-borne gene variants (Fig 1). When using SNPs to assess relationships (Fig 1B), some tight clustering can be observed for pairs or triplets of isolates carrying *bla*_CTX-M-1_ (64.1 and 81.1 with 30 SNPs, as well as 56.2 and 276.2 with 46 SNPs), *bla*_CTX-M-55_ (136.2 and 268.2, as well as 101.3 with 23 to 43 SNPs, 176.1 and 154AHL with 23 SNPs) and *bla*_CTX-M-65_ (98.1 and 202.1 with 37 SNPs).

#### Plasmid sequences and relationships

Nine genomes were assembled successfully with Unicycler, and the remaining six were completed using Flye. An average of 59x coverage was obtained with long reads, and a minimum of 48x with short reads. Closed plasmids were verified with reference mapping using long reads.

**Fig 2:**
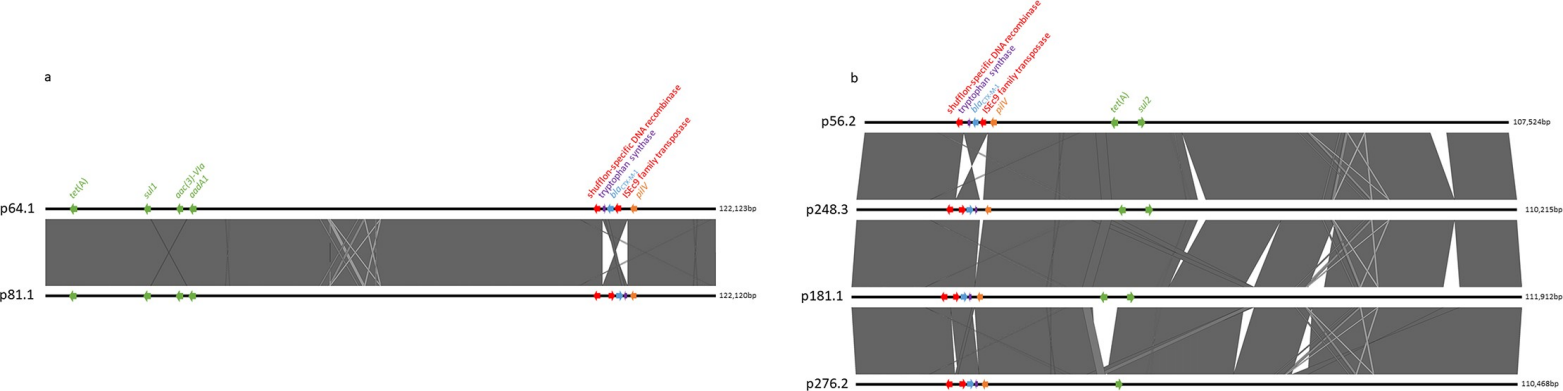
Sequence alignments of plasmids carrying the *bla*_CTX-M-1_ gene. **a:** Sequence alignment of two IncI1 *bla*_CTX-M-1_ plasmids of unknown pMLST type. **b:** Sequence alignment of four IncI1 *bla*_CTX-M-1_ plasmids of pMLST type 3. The names of the plasmids correspond to the isolates they originate from (Table 4). Only genes and genetic elements mentioned in the text are shown. Resistance genes other than *bla*_CTX-M-1_ (in blue) are labelled in green.

All *bla*_CTX-M-1_ plasmids belonged to the IncI1 group but carried varied combinations of resistance genes. Further analysis of the *bla*_CTX-M-1_ plasmids showed that they varied in size between ~107 kbp and ~122 kbp. They belonged to two different pMLST types (type 3 and a new yet undescribed type), which correlated broadly with plasmid sizes (Table 4). Sequence alignments of the two plasmids from the new pMLST type (isolates 64.1 and 81.1, Fig 2A) showed strong similarities. They both carried a *tet*(A) gene and a class 1 integron with two aminoglycoside resistance cassettes and its associated *sul1* gene. The only major difference between these two plasmids was an inversion in the region containing *bla*_CTX-M-1_ (Fig 2A), flanked on one side by the recombinase and on the other by the *pilV* gene of the R64 shufflon system [37]. These two plasmids were found in two genetically related isolates of the same ST3258 (Table 4, Fig 1). The four pMLST type 3 plasmids also showed relatively similar structures, but with multiple regions of recombination (Fig 2B). One of the major recombination sites among these plasmids contained an inversion of the *bla*_CTX-M-1_ region between *pilV* and a shufflon recombinase gene (Fig 2B) similar to the one described above. A *tet*(A) gene was present in another recombination site in three of these four plasmids. This region was flanked on one side by a more conserved region containing a *sul2* gene in all four plasmids. No class 1 integron was present in these pMLST type 3 plasmids. They were distributed among isolates of two different unrelated STs, including the widespread poultry-associated ST117 (Table 4, Fig 1).

The other plasmids belonged to the IncF complex, with a variety of IncFIA, IncFIB, IncFIC, IncFII and IncR combinations (Table 4). While the two *bla*_CTX-M-65_ plasmids presented with the same replicons and antimicrobial resistance genes, the five *bla*_CTX-M-55_ plasmids showed a variety of replicon markers and antimicrobial resistance genes combinations (Table 4).

**Fig 3:**
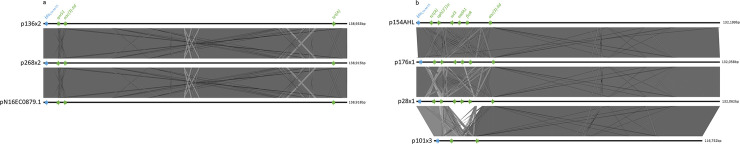
Sequence alignments of plasmids carrying the *bla*_CTX-M-1_ gene. **a:** Sequence alignment of two IncFIA/IncFIB/IncFII *bla*_CTX-M-55_ plasmids. **b:** Sequence alignment of the four IncFIB/IncFIC(FII) *bla*_CTX-M-55_ plasmids. The names of the plasmids correspond to the isolates they originate from (Table 4). Resistance genes other than *bla*_CTX-M-55_ (in blue) are labelled in green.

The six *bla*_CTX-M-55_ plasmids varied in size between ~117kbp and ~139 kpb and showed two IncF replicon combinations (Table 4). The two IncFIA/IncFIB/IncFII plasmids had both the same alleles in the IncF pMLST scheme and sequences alignment showed a very high level of similarity without any significant area of recombination (Fig 3A). They were both located in isolates belonging to the ST58 type (Fig 1). These two plasmids were almost identical to plasmid pN16EC0879-1 from an *E*. *coli* isolate of the same ST58 found in ground turkey in the USA (Table 5; Fig 3A). The four antimicrobial resistance determinants of these plasmids were all clustered in the same region and included genes for resistance to three antimicrobials (extended-spectrum cephalosporins, fluoroquinolones, and gentamicin) considered as critically important for human medicine [1]. The core genome of the isolate from the USA differed from the two isolates of this study by only 44 and 51 SNPs, respectively. The four other IncFIB/IncFIC plasmids carrying *bla*_CTX-M-55_ had partially similar but not always identical sets of alleles in the IncF pMLST scheme (Table 4). All of them carried the *cma* and *cba* genes for colicin M and B as well as *cmi* and *cbi* for the corresponding colicin inhibitors. Sequence alignments showed that three of these plasmids (from isolates 28.1, 176.1 and 154AHL) had very similar sizes and structure (Fig 3B), with the same combination and arrangement of seven antimicrobial resistance genes. The fourth plasmid of this group also showed structural similarities with the three former ones but was smaller (~117kbp instead of ~132–139 kbp). It lacked the atypical class 1 integron-associated region containing the *floR*, *aadA*, and *sul3* genes found in the others (Fig 3B). Although the isolates from which these four plasmids originate show some loose genetic relationships (Fig 1), they belong to two different STs (including the widespread ST10) and the plasmid structures observed don’t seem to correlate with these STs and putative genomic relationships between isolates. The isolates carrying these four plasmids are completely unrelated to the two other ST58 isolates carrying *bla*_CTX-M-55_ discussed earlier.

**Fig 4:**
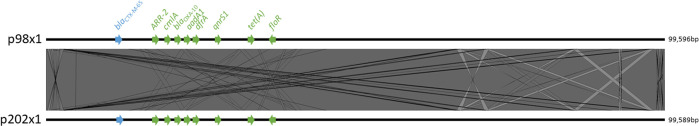
Sequence alignment of plasmids carrying the *bla*_CTX-M-65_ gene. The names of the plasmids correspond to the isolates they originate from (Table 4). Resistance genes other than *bla*_CTX-M-55_ (in blue) are labelled in green.

The two *bla*_CTX-M-65_ plasmids had the same pMLST allele combinations (with a new FIA allele closely related to A13 and A18) and were almost identical in size (Table 4), structure and sequence (Fig 4); they carried nine genes for resistance to six different antimicrobial classes, including three (extended-spectrum cephalosporins, fluoroquinolones, and aminoglycosides) considered critically important for human medicine [1]. The isolates carrying these plasmids were closely related (37 SNPs) and belonged to ST683, a type already associated previously with *bla*_CTX-M-65_ in *E*. *coli* from Canadian beef cattle [12]. The *bla*_CTX-M-65_ plasmids from isolate 98.1 from this study and from a representative Canadian beef isolate could not be transferred by conjugation under any combination of the conditions tested (i.e. using mating on solid or in liquid media at room temperature or at 37°C).

**Fig 5:**
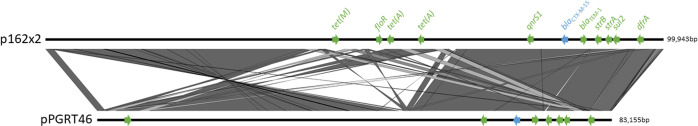
Sequence alignment of a plasmid carrying the *bla*_CTX-M-15_ gene and the most closely related plasmid sequence available on GenBank. The names of the plasmids correspond to the isolate it originates from (Table 4). Resistance genes other than *bla*_CTX-M-15_ (in blue) are labelled in green.

Finally, the map of the only *bla*_CTX-M-15_ plasmid sequenced in this study is presented in Fig 5. Although its sequence shows significant similarity with other *bla*_CTX-M-15_ plasmids such as pPGRT46 (Fig 5), differences are present in its structure including a major inversion and the insertion of an additional antimicrobial resistance region resulting in a duplication of the *tet*(A) gene.

## Discussion

The results of this study show that, two years after the withdrawal of ceftiofur in the Canadian turkey industry [3, 4], ESC-resistant *Enterobacterales* can still be isolated in two thirds of pooled fecal samples from turkey and in a majority (82%) of the sentinel farms examined. Although a few *K*. *pneumoniae*, *P*. *mirabilis*, and *E*. *cloacae* isolates were recovered, the vast majority of ESC-resistant isolates obtained from these samples through enrichment were *E*. *coli*. However, the sampling strategy used to select isolates on plates was heavily biased toward *E*. *coli* and other lactose fermenters and may have resulted in an underestimated prevalence for *Enterobacterales* other than *E*. *coli*, in particular for lactose-negative species (e.g. *P*. *mirabilis*). Nevertheless, the low recovery rate of ESC-resistant *K*. *pneumoniae* and *E*. *cloacae* (both lactose positive) strongly suggest that *E*. *coli* represents the main reservoir of ESC resistance determinants among fecal *Enterobacterales* in turkey flocks.

Not surprisingly, the proportion of samples positive for ESC-resistant *E*. *coli* was higher (65%) when using enrichment cultures and selective media than when testing a single colony after plating samples directly on non-selective media (5%; [38]). This difference clearly illustrates that only a small fraction of the *E*. *coli* present in fecal samples are indeed resistant to ESCs. This stresses the need for the additional use of selective media in epidemiological studies assessing the presence or persistence of ESC-resistant bacteria at the animal or farm level. The prevalence of ESC resistance in *E*. *coli* isolates from diagnostic submissions (4%) was not alarmingly high and in the same range as for generic fecal isolates (5%). It was similar to the prevalence found in poultry in other countries where the use of cephalosporins has recently been drastically reduced [39].

Except for the generic fecal *E*. *coli* isolates collected by CIPARS, *bla*_CMY_ was the main ESC resistance determinant in both *E*. *coli* from fecal enrichment cultures and in those from diagnostic submissions. The same was true for *S*. *enterica* in which only *bla*_CMY_ could be detected. This is consistent with the situation in other farm animals in Canada [11]. In addition, further characterization of a subsample of these *bla*_CMY_ genes confirmed that they encoded the typical CMY-2 variant and were located on plasmids of replicon types (IncI and IncK) known to be frequently associated with this AmpC β-lactamase in bacteria from farm animals in Canada [8]. Interestingly, none of the plasmids from our subsample belonged to the replicon type IncA/C, which is also known to frequently carry *bla*_CMY_ in *E*. *coli* and *S*. *enterica* from animals [7, 8]. The second most frequent β-lactam resistance gene found in *E*. *coli* from fecal enrichment cultures was *bla*_TEM_. This gene was generally found together with *bla*_CMY_, *bla*_CTX-M_ or *bla*_SHV_. It was also of a variant (*bla*_TEM-1B_) not responsible for the ESBL phenotype observed in the only two isolates in which it was not accompanied by one of these three other ESC resistance genes. We therefore assume that most of the *bla*_TEM_ genes found in this study were not encoding ESBLs. The third most frequent ESC-resistance gene found was *bla*_CTX-M_, a relative newcomer on the ESC resistance scene in farm animals in Canada [9, 12]. Whole genome sequencing showed that at least four variants of this gene also found in bacteria from other farm animal species [16] as well as in bacteria from humans and urban wastewaters in Canada [12, 40] have made their way into *E*. *coli* from turkeys (Table 4). No other published study on *bla*_CTX-M_ genes in *Enterobacterales* from turkey feces or from clinical samples from Canada and the USA is currently available for comparison. However, a recent publication from the USA showed that among six *E*. *coli* isolates from turkey meat, two carried the same plasmid-borne *bla*_CTX-M_ variants (i.e. *bla*_CTX-M-1_ and *bla*_CTX-M-15_) as in the present study [41]. The least frequent ESC resistance gene detected in *E*. *coli* from enrichment cultures was *bla*_SHV_. This contrasts with isolates recovered by CIPARS from the same samples by direct plating on non-selective media and without enrichment. Although the number of ESC-resistant isolates obtained without selective media was small, the proportion of *bla*_SHV_-positives among them is significantly higher than after enrichment and use of selective media (p-value = 0.0007 with Fisher’s exact test). Despite this difference in apparent prevalence, the SHV variants of the isolates recovered both by CIPARS and with our selective media were all SHV-2 and SHV-2a. This is in agreement with results of an earlier study in which the same two SHV variants were the only ones found in poultry in Canada [13]. *Enterobacterales* with these two SHV variants have relatively low MICs for ESCs and the median ceftazidime and ceftriaxone MICs for *E*. *coli* isolates producing these variants is precisely in the concentration range used for our selective media [13]. Therefore, it is likely that the cephalosporins used in our selective media may have favoured the growth of isolates with other ESC resistance mechanisms, in particular CTX-M variants with their higher cefotaximase activity [42, 43] thus leading to an underestimation of the *bla*_SHV_ genes prevalence. We assume that, if present, bacteria producing other SHV variants with higher MICs would however not be at such a strong disadvantage and would be recovered from enrichment cultures more frequently than SHV-2 and SHV-2a. This is supported by the recovery of two *K*. *pneumoniae* isolates with an ESBL phenotype in this study which produced a SHV-148, a variant associated with higher ESC MICs [44].

Among the 17 *bla*_CTX-M_-positive *E*. *coli* isolates sequenced, three pairs of genetically closely related isolates presented with very similar plasmids within each pair (i.e. ST3258 with *bla*_CTX-M-1_, ST58 with *bla*_CTX-M-55_, and ST683 with *bla*_CTX-M-65_). To the best of our knowledge, only the combination of ST58 and *bla*_CTX-M-55_ has been described in the literature to date [45], thus suggesting a possible infrequent occurrence of these strains and plasmids combinations. This supports the presence of epidemiological links between the isolates of each pair. The isolate within each pair originated from different farms and vertical transmission between parent flocks or hatcheries and grower farms is the most likely explanation for these findings. As illustrated by the strong similarities observed between our two ST58 isolates carrying identical *bla*_CTX-M-55_ plasmids and an isolate recovered from ground turkey meat in the USA, epidemiological links between ESC-resistant isolates from live birds may possibly extend further downstream into the food chain. These findings show that the expansion of clonal lineages carrying *bla*_CTX-M_ genes is a significant part of the spread of ESBLs among farms and possibly along the turkey production continuum. This conclusion is also supported by the presence of a pair of related *E*. *coli* isolates of the same ST683 carrying a *bla*_CTX-M-65_ variant. This same *bla*_CTX-M-65_-positive clonal lineage was also found among geographically unrelated beef cattle in Alberta [12]. The lack of *in vitro* conjugative transfer of the associated *bla*_CTX-M-65_ plasmids from representative isolates from both beef and turkey under a variety of conditions suggests that clonal expansion can play an important role in the spread of at least some CTX-M ESBLs, not only in turkey, but in Canadian animal populations at large. Although less obvious (i.e. isolates less tightly clustered and similar plasmids with more extensive recombination), correlations between plasmid structure and chromosomal genotype were also present among isolates belonging to more frequent and globally widespread clonal lineages, such as ST10 and ST117 [46, 47]. Thus, clonal evolution over longer time periods and a larger geographical scale and operational factors inherent to the turkey sector such as international and interprovincial exchange of turkey products (e.g., breeder stocks, hatching eggs and poults) may also be at play in the spread of CTX-M ESBLs in the turkey industry. The pMLST type 3 plasmid carrying *bla*_CTX-M-1_ is known to be an epidemic plasmid which has spread across a wide variety of bacterial strains and animal hosts [48]. Its presence in related (the ST117 isolates 56.2 and 276.2 with 46 SNPs) as well as distantly related isolates (the ST115 isolate 248.3 with 65633 and 65647 SNPs when compared to the two preceding ST117 isolates) in our study is a clear illustration of the role of horizontal gene transfer in the spread of at least some *bla*_CTX-M_ plasmids in addition to the clonal expansions mentioned above.

While only three of the 18 *bla*_CMY-2_ plasmids investigated carried genes for resistance to other antimicrobials, all 15 *bla*_CTX-M_ plasmids did (one with eight additional antimicrobial resistance genes). Resistance to antimicrobial agents frequently used to prevent and treat diseases in poultry was encoded by these 15 plasmids. This includes resistance to tetracyclines (n = 14) and sulfonamides (n = 10) which are both still used in turkeys [38]. This suggests that co-selection of ESC resistance plasmids could possibly occur through the use of these antimicrobials. A similar situation has been encountered in Canadian chickens, where use of spectinomycin seems to have selected resistance to other antimicrobials [17]. This included among others resistance to gentamicin which was encoded by a gene collocated on the same plasmids as the *aadA* resistance gene for spectinomycin [9]. The more frequent multidrug resistance encoded by *bla*_CTX-M_ plasmids observed here suggests that these plasmids may be at an advantage in comparison to the current *bla*_CMY-2_ plasmids. Eight of the 15 sequenced *bla*_CTX-M_ plasmids carried a gentamicin resistance gene. This may have contributed to their maintenance, as gentamicin was still frequently injected into poults at the time of this study [38]. Incidentally, the *qnrS1* gene for reduced susceptibility to the critically important fluoroquinolones was present in five of the sequenced plasmids and may be maintained in similar ways.

## Conclusions

Our results show that ESC-resistant *Enterobacterales* were still frequently present in Canadian turkeys two years after the cessation of ceftiofur use. Resistance to these antibiotics is still caused in majority by CMY-2. However, a variety of CTX-M variants are now also widespread in *Enterobacterales* from Canadian turkeys. Both local clonal expansion of CTX-M producing *E*. *coli* through the turkey meat production chain and horizontal gene transfer of globally epidemic plasmids seem to contribute to the spread of plasmids encoding CTX-M in fecal bacteria of Canadian turkey populations. The frequent multidrug resistance nature of these plasmids suggests that despite the recent changes in antimicrobial use policy in the farming industry in this country, co-selection with antimicrobial agents not critical for human medicine could result in further lingering of ESCs resistance determinants, in particular of *bla*_CTX-M_ genes.
